# Supplementary material for: Multi-stage bioengineering of a layered oesophagus with in vitro expanded muscle and epithelial adult progenitors
Source: Nat Commun. 2018 Oct 16;9:4286. doi: 10.1038/s41467-018-06385-w (PMC6191423; doi:10.1038/s41467-018-06385-w)
Supplement: Supplementary file 1 — Supplementary Information [file 41467_2018_6385_MOESM1_ESM.pdf]

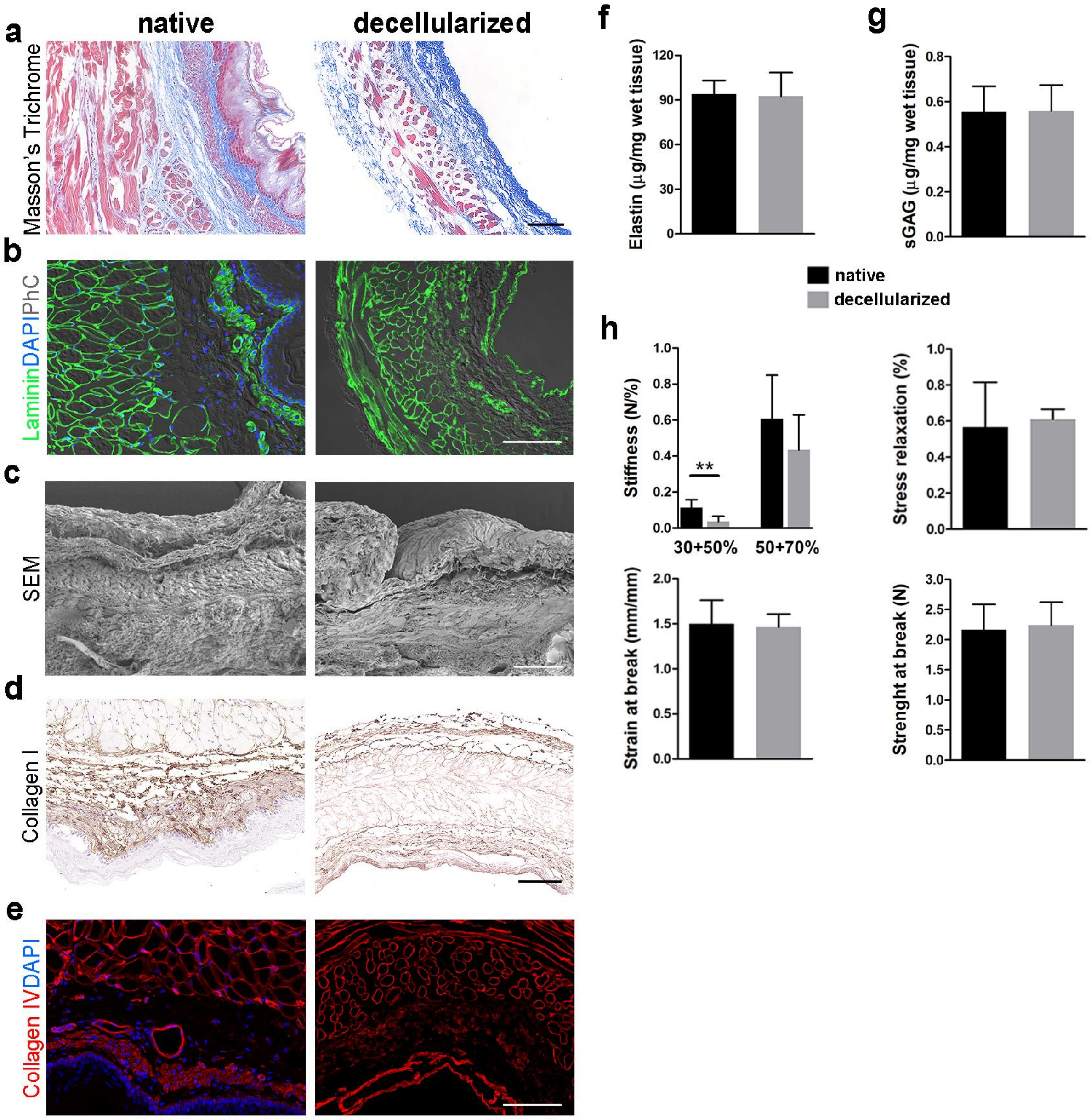

Supplementary Figure 1. Characterization of decellularized rat oesophageal scaffolds.

(**a**) Masson's trichrome on sections of native oesophagus and decellularized scaffold. Scale bar: 100µm. (**b**) Laminin and DAPI immunostaining (with phase contrast). Scale bar: 100µm. (**c**) Scanning electron microscopy (SEM) images of oesophagi before and after decellularization. Scale bar: 100µm. Immunohistochemistry for collagen I (**d**) and immunofluorescence for collagen IV (**e**) in native and decellularized oesophagi. Scale bar: 100µm. (**f-g**) Quantification of elastin (g) and glycosaminoglycans (h) in native and decellularized oesophagi (n=3). (**h**) Bio-mechanical analysis of native and decellularized oesophagi: analysis of stiffness at 30+50% of strain and 50+70% of strain, stress relaxation (residual stress after relaxation), ultimate strain at break and ultimate tensile strength at break (n=5; **p=0.0087; t-test).

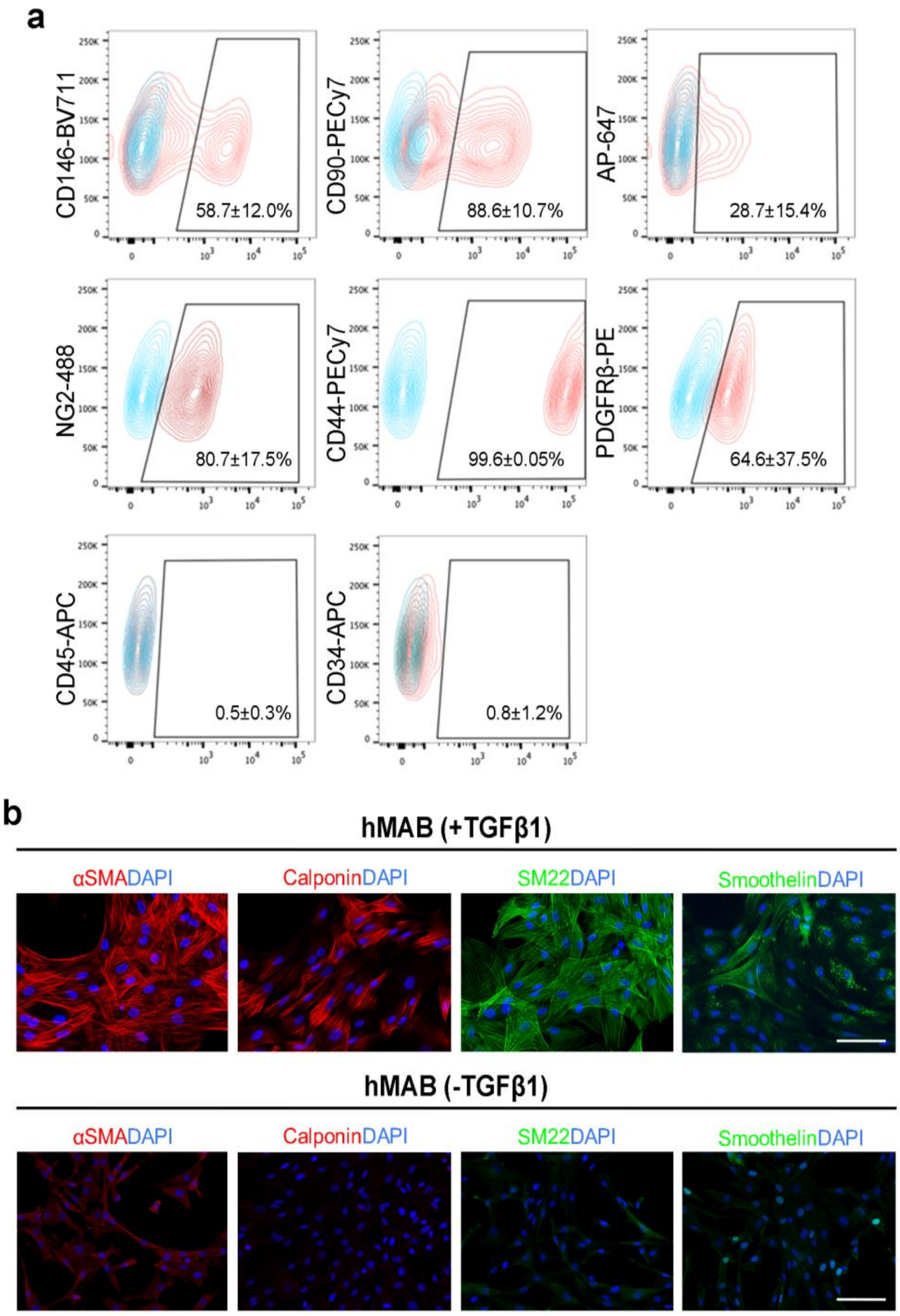

Supplementary Figure 2. FACS characterization and smooth muscle differentiation in 2D culture of expanded hMAB.

(**a**) Representative FACS analysis of expanded hMAB for CD146, CD90, AP, NG2, CD44, PDGFRβ, CD45 and CD34. Markers are indicated with fluorochrome used. Percentage of positive cells is indicated in the gate as mean±standard deviation (n=3÷4). (**b**) hMAB cultured in 2D with TGFβ1 for 7 days showing smooth muscle differentiation by immunostaining for αSMA, calponin and SM22 (top). Mature smooth muscle differentiation was obtained with 2 weeks of TGFβ1 as shown by positivity for smoothelin. Immunostaining for smooth muscle markers was negative in cell cultured without TGFβ1 (bottom – undifferentiated control cells). Nuclei were stained with DAPI. Scale bar: 100µm.

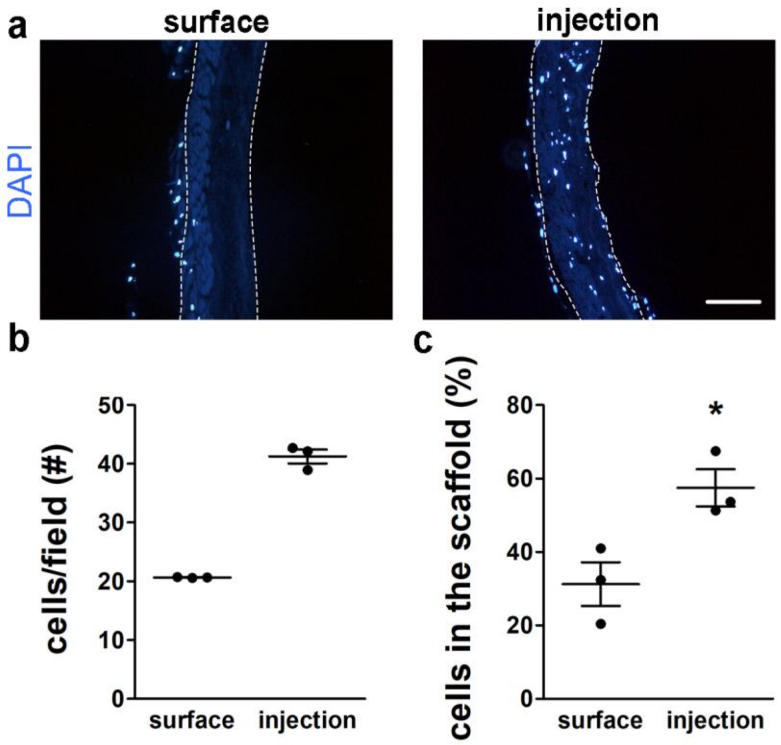

Supplementary Figure 3. Comparison of surface versus multiple-injection seeding of hMAB into decellularized oesophageal scaffolds.

(**a**) Representative images of DAPI staining on sections of decellularized scaffolds seeded with hMAB using surface or multiple-injection seeding and cultured in static for 4 days. Dotted line indicates the edges of the scaffold. Scale bar: 100µm. (**b-c**) Total number of cells per area (**b**) and percentage of the number of cells in layers of the scaffold underneath the surface (**c**) in scaffolds seeded with surface seeding or multiple-injections of cells. Data: mean±SEM (n=3; *p=0.0283; t-test).

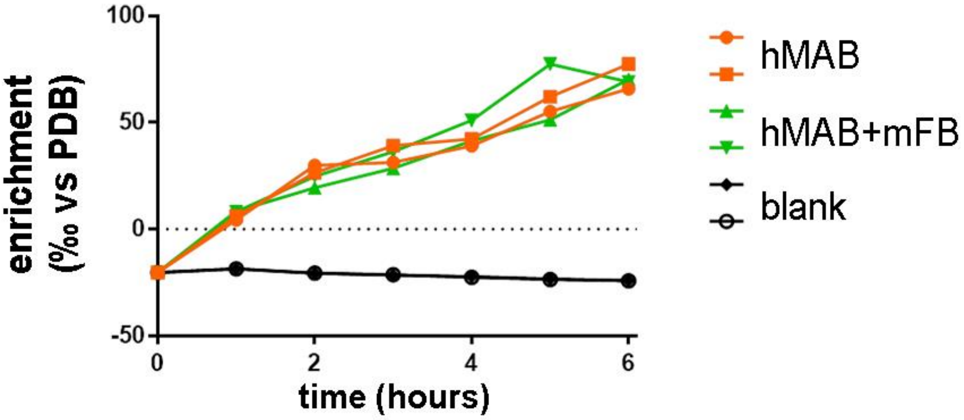

Supplementary Figure 4. 2D culture controls for oxidative metabolic analysis with ^{13}C-glucose. Oxidative metabolism (^{13}C-glucose oxidation assay) measured as $^{13}CO_2$ production in cultured media sampled every hour from 2D cultures of hMAB and hMAB+mFB in presence of TGFβ1.

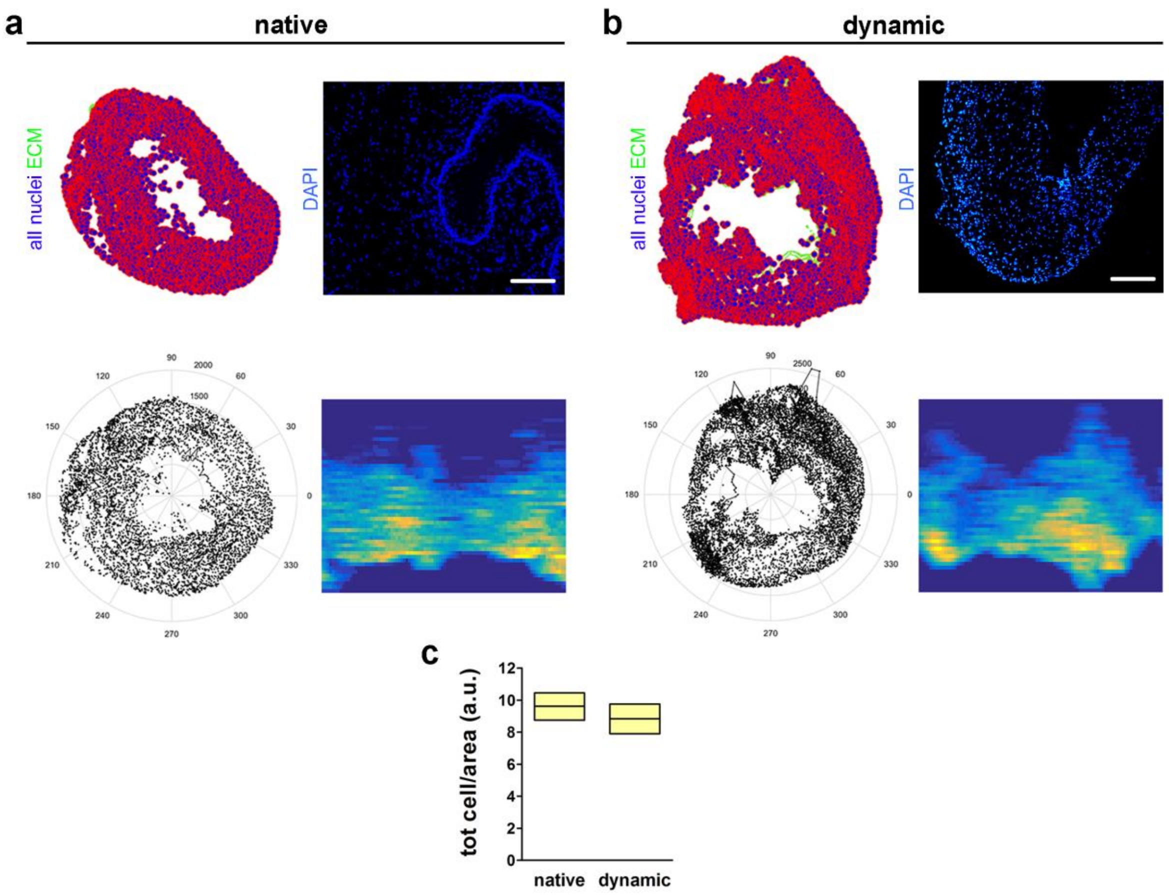

Supplementary Figure 5. Cell distribution analysis of native oesophagi and re-populated decellularized scaffolds after dynamic culture.

(**a,b**) Cell distribution maps, representative DAPI staining, polar distribution maps and cell density maps obtained from DAPI-stained sections of a native rat oesophagus without mucosa (**a**) and a scaffold seeded with hMAB+mFB+mNCC and cultured in dynamic condition for 11 days (**b**). Scale bar: 100μm. (**c**) Total number of cells per field identified in sections of native oesophagi and recellularized scaffolds stained for DAPI. Data: mean±min to max (n=1).

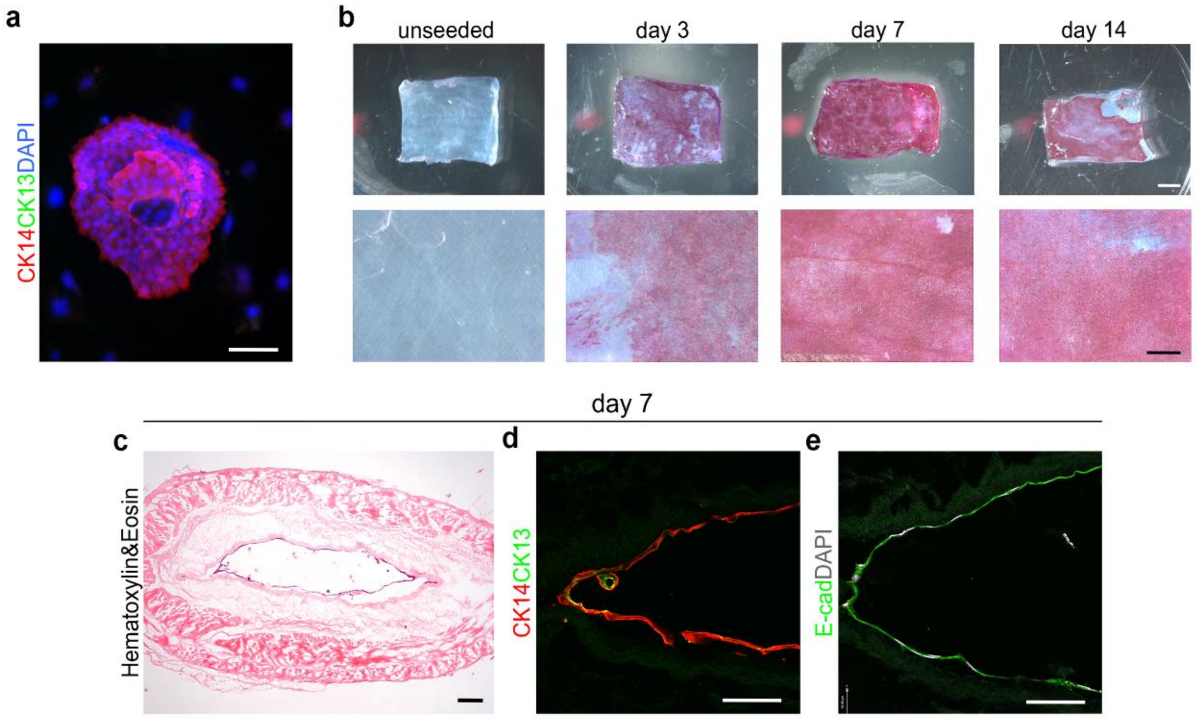

Supplementary Figure 6. ROEC growth and distribution on slit-open and tubular decellularized scaffold.

(**a**) Immunofluorescence of ROEC for the expression of CK14 and CK13 at day 4 in culture. Scale bar: 100µm. (**b**) Images of MTT colorimetric assay on slit-open scaffolds seeded with ROEC and cultured in air-liquid interphase condition for up to 14 days. Viable cells are coloured in purple. Scale bar: 2mm (top) and 250µm (bottom). (**c**) Hematoxylin and eosin staining of sections of tubular scaffold seeded with ROEC and cultured in static for 7 days. Scale bar: 200µm. (**d-e**) Immunostaining for CK13 and CK14 (d) and E-cadherin and DAPI (e) on sections of tubular scaffold seeded with ROEC and cultured in static for 7 days. Scale bar: 84µm.

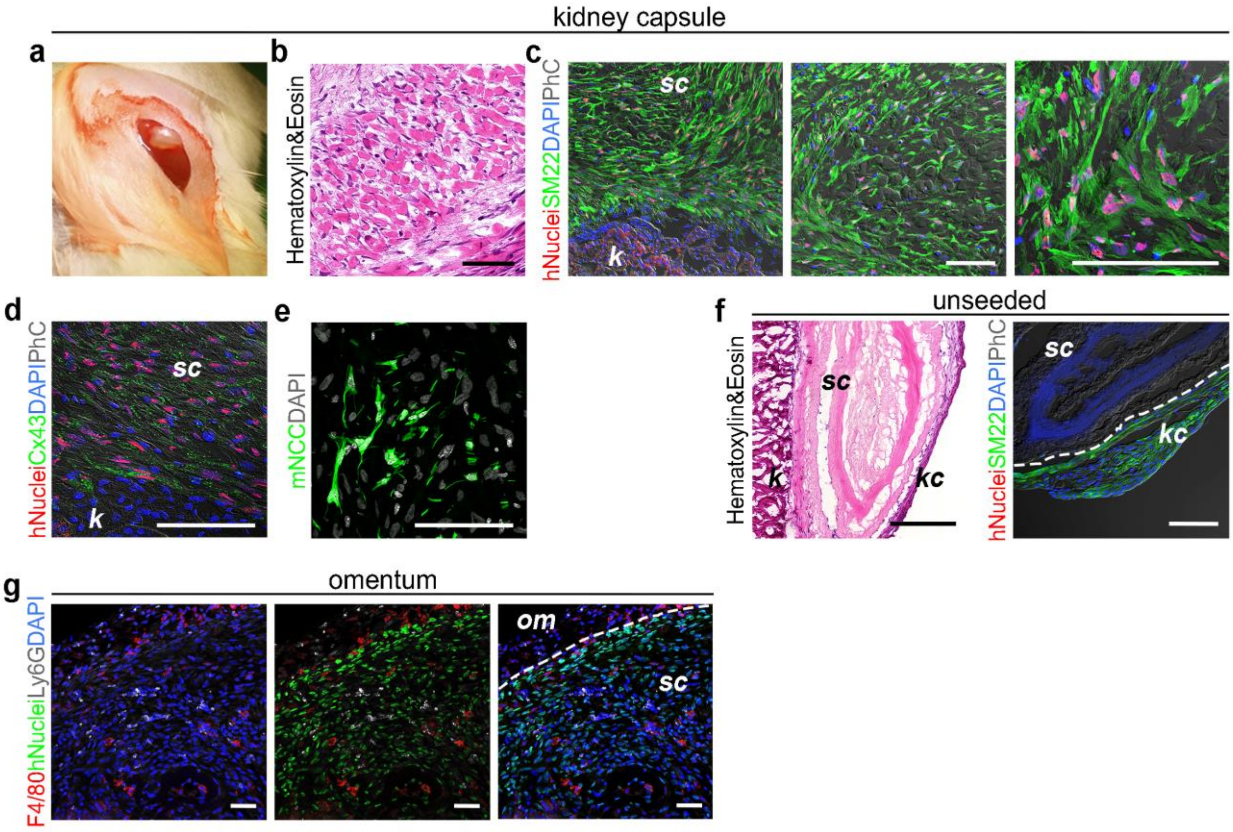

Supplementary Figure 7. Analysis of re-populated scaffolds post-implantation under the kidney capsule and inflammatory reaction in omental implants.

Analysis of scaffolds seeded with hMAB+mFB+mNCC, cultured in dynamic for 11 days, implanted under the kidney capsule of NSG mice and harvested after 1 week. All images show the *muscularis externa* of the re-populated scaffolds. (**a**) Picture of the surgical implantation of the re-populated scaffold. (**b**) Hematoxylin and eosin staining. Scale bar: 100µm. (**c**) Immunofluorescence staining for hNuclei, SM22 and DAPI (with phase contrast). Scale bar: 100µm. (**d**) Immunofluorescence staining for hNuclei, Connexin43 and DAPI (with phase contrast). K: kidney; sc: scaffold. Scale bar: 100µm. (**e**) Immunofluorescence staining for GFP (indicating the mNCC) and DAPI. Scale bar: 100µm. (**f**) Images of unseeded scaffolds 7 days after implantation under the kidney capsule. Hematoxylin and eosin staining (left – scale bar: 250µm) and immunostaining for hNuclei, SM22 and DAPI (with phase contrast) (right – scale bar: 100µm). Sc: scaffold; k: kidney; kc: kidney capsule; dotted line indicates the separation line between kidney capsule and scaffold. **(g)** Representative images of immunofluorescence for F4/80, Ly6G, hNuclei and DAPI in scaffolds seeded with hMAB+mFB+mNCC, 7 days post-implantation in the omentum of NSG mice. Scale bar: 50µm. Sc: scaffold; om: omentum; dotted line indicates the separation line between omentum and scaffold.

Supplementary Table 1. Antibodies and dilutions for immunofluorescence and immunohistochemistry.

Antibody	Host	Company	Cat. number	Dilution
Calponin	Mouse	Sigma	C2687	1:1000
Caspase3	Rabbit	Cell Signalling	9661	1:100
CK13	Mouse	Abcam	ab16112	1:100
CK14	Rabbit	BioLegend	PRB-155P	1:800
Collagen I	Mouse	Abcam	ab6308	1:1000
Collagen IV	Rabbit	Abcam	ab6586	1:1000
Connexin43	Rabbit	Abcam	ab11370	1:100
E-cadherin	Mouse	BD Transduction Laboratories	610181	1:100
F4/80	Rat	BioLegend	123110	1:100
GFP	Rabbit/Chick	Life	ab6455/ab13970	1:500
hNuclei	Mouse	Millipore	MAB1281	1:200
Ki67	Rabbit	Abcam	ab15580	1:400
Laminin	Rabbit	Abcam	ab11575	1:400
Ly-6G	Rat	BioLegend	127610	1:200
NG2	Rabbit	Abcam	ab83178	1:100
p63	Mouse	Abcam	ab735	1:50
PanCytokeratin	Rabbit	Invitrogen	18-0059	1:100
PDGFRβ	Rabbit	Abcam	ab32570	1:100
Phalloidin-488		Life Technology	A12379	1:100
S100	Rabbit	Dako	Z031101	1:500
SM22	Rabbit	Abcam	ab14106	1:1000
Sox10	Goat	Santa Cruz	Sc-365692	1:500
TCF-4	Mouse	Millipore	05-511	1:100
TuJ1	Mouse	Covance	MMS435P	1:500
Vimentin	Mouse	Abcam	ab20346	1:100
vWF	Rabbit	Abcam	ab9378	1:100
αSMA	Mouse	Abcam	ab7817	1:50
Anti-mouse 568	Goat	Invitrogen	A1104	1:1000
Anti-rabbit 488	Goat	Invitrogen	A11008	1:350
Anti-goat 594	Donkey	Invitrogen	A11058	1:500
Anti-chicken 488	Goat	Invitrogen	A11039	1:500

Supplementary Table 2. FACS antibodies and dilutions.

Antibody	Wavelength	Company	Cat. number	Dilution
CD146	BV711	BD	536186	1:200
CD56	BV395	BD	563554	1:200
CD90	PE-Cy7	BD	561558	1:200
AP	647	BD	561500	1:100
NG2	488	BD	562413	1:200
CD44	PE-Cy7	BioLegend	103030	1:200
PDFGRβ	PE	BioLegend	323606	1:50
CD45	APC	BioLegend	304011	1:200
CD34	APC	Miltenyi	130090954	1:20
